# The Model of Gamification Principles for Digital Health Interventions: Evaluation of Validity and Potential Utility

**DOI:** 10.2196/16506

**Published:** 2020-06-10

**Authors:** Mark Floryan, Philip I Chow, Stephen M Schueller, Lee M Ritterband

**Affiliations:** 1 Department of Computer Science University of Virginia Charlottesville, VA United States; 2 Department of Public Health Sciences University of Virginia Charlottesville, VA United States; 3 University of California Irvine, CA United States

**Keywords:** gamification, internet interventions, eHealth, mHealth, digital health

## Abstract

**Background:**

Although gamification continues to be a popular approach to increase engagement, motivation, and adherence to behavioral interventions, empirical studies have rarely focused on this topic. There is a need to empirically evaluate gamification models to increase the understanding of how to integrate gamification into interventions.

**Objective:**

The model of gamification principles for digital health interventions proposes a set of five independent yet interrelated gamification principles. This study aimed to examine the validity and reliability of this model to inform its use in Web- and mobile-based apps.

**Methods:**

A total of 17 digital health interventions were selected from a curated website of mobile- and Web-based apps (PsyberGuide), which makes independent and unbiased ratings on various metrics. A total of 133 independent raters trained in gamification evaluation techniques were instructed to evaluate the apps and rate the degree to which gamification principles are present. Multiple ratings (n≥20) were collected for each of the five gamification principles within each app. Existing measures, including the PsyberGuide credibility score, mobile app rating scale (MARS), and the app store rating of each app were collected, and their relationship with the gamification principle scores was investigated.

**Results:**

Apps varied widely in the degree of gamification implemented (ie, the mean gamification rating ranged from 0.17≤m≤4.65 out of 5). Inter-rater reliability of gamification scores for each app was acceptable (κ≥0.5). There was no significant correlation between any of the five gamification principles and the PsyberGuide credibility score (*P*≥.49 in all cases). Three gamification principles (supporting player archetypes, feedback, and visibility) were significantly correlated with the MARS score, whereas three principles (meaningful purpose, meaningful choice, and supporting player archetypes) were significantly correlated with the app store rating. One gamification principle was statistically significant with both the MARS and the app store rating (supporting player archetypes).

**Conclusions:**

Overall, the results support the validity and potential utility of the model of gamification principles for digital health interventions. As expected, there was some overlap between several gamification principles and existing app measures (eg, MARS). However, the results indicate that the gamification principles are not redundant with existing measures and highlight the potential utility of a 5-factor gamification model structure in digital behavioral health interventions. These gamification principles may be used to improve user experience and enhance engagement with digital health programs.

## Introduction

There is substantial interest in understanding how gamification can improve electronic health (eHealth) and mobile health (mHealth) interventions [1-3], yet significant gaps in the literature remain. Metareviews of gamification strategies within behavioral interventions summarize the work in this area [4,5] but often focus on the mechanics employed (eg, badges, leaderboards, etc) and neglect other potentially important factors (ie, the context of the app). In addition, individual studies of gamification of digital health interventions typically present results as general game vs control studies [6,7] or focus more on the qualitative and subjective aspects of gamification [8]. As gamification techniques are intertwined with other intervention components, their validity and incremental impact are relatively unknown.

The model of gamification principles for internet interventions [9] was developed to present a unifying, theory-driven set of five gamification principles (Textbox 1) that can be used in the building and testing of digital health interventions. There is no widespread agreement on how the principles of gamification should be applied to eHealth/mHealth interventions. Although the detailed justification for the development of these principles is beyond the scope of this paper, additional information can be found in the prior study on which this one is based [9]. These principles, which were extracted from several well-known gamification models, represent independent and actionable items regarding the application of gamification (see the study by Floryan et al [9] for details). The model is composed of five separate yet interrelated constructs: meaningful purpose, meaningful choice, supporting player archetypes, feedback, and visibility. Textbox 1 summarizes the five gamification principles and provides a short description of each principle. The principles provide concrete and measurable descriptions of gamification while focusing on the context, goals, and attitudes of users of the program. These principles provide a descriptive framework for measuring both the presence and quality of gamification implementation. Although these have been well defined, empirical validation is a necessary next step.

**Summary of principles of gamification.**

Gamification principle and description:

- Meaningful purpose: The app presents goals that align with the user’s motivations and interests
- Meaningful choice: The app gives users agency over how they achieve their goals
- Supporting player archetypes: Mechanics in the app leverage individual user and player characteristics
- Feedback: The app communicates how user actions affect progress
- Visibility: The app makes clear to users the amount of progress made and how much more is needed

The proposed gamification principles encourage developers to separate the idea of typically considered gamification mechanics (eg, points, badges, and leaderboards) from the purpose of those mechanics (eg, motivating the purpose, increasing user choice, supporting player archetypes, etc). In this way, the model encourages researchers to consider the mechanics of gamification and how those relate to the underlying motivational affordances of the user. It also provides researchers with a framework for implementing these mechanics within the technology-based interventions they create. In behavioral science, several attempts have been made to specify various behavior change strategies [10] and call for increased use of evidence-based behavior change techniques within research and commercially developed products and interventions [11]. However, there have been few efforts to map behavior change principles to design features that could be implemented by researchers. The principles of gamification attempt to address this mapping by providing actionable principles contextualized by known behavior change principles for internet interventions (details regarding this mapping can be found in the study by Floryan et al [9]). The specification of these principles can facilitate research and lead to a better understanding of these mechanisms and how they can best be used. The goals of this study were to understand the validity and reliability of this 5-factor gamification model. 

## Methods

### Overview

Mobile- and Web-based interventions were selected from PsyberGuide, a nonprofit endeavor that aims to provide consumers with information to aid in selecting different types of mental health apps. PsyberGuide conducts independent and unbiased reviews of mental health apps and evaluates products on three dimensions: credibility, user experience, and privacy and data security. PsyberGuide has evaluated over 200 mental health apps and is viewed as a useful standard for determining the quality of apps on these various metrics [12-14]. Given the variety of apps reviewed on PsyberGuide and the variance in scores of credibility and user experience of these apps, it provided a useful point of comparison to evaluate the gamification assessment. Specifically, the credibility and user experience scores (see the *Measures* section for more detail) for each app were useful to compare the presence of gamification with these other metrics. Apps were selected (Table 1) in February 2018 and evaluated for the presence and implementation of the five internet intervention gamification principles (Textbox 1). They were selected using the following criteria: (1) presence on the PsyberGuide listing, (2) free to use or provided core content free of charge, (3) broadly applicable to the general population in the domain of behavioral and mental health, and (4) available through at least one of the Apple App Store, Android Store, or a Web browser (eg, website based). A total of 133 trained independent raters and undergraduates enrolled in a college-level human-computer interaction (HCI) course served as judges. The raters were all aged between 18 and 22 years and majoring in computer science or a related field (eg, systems engineering, computer engineering). Many of the raters were double majoring in a related field (eg, cognitive science, psychology, etc). To obtain adequate interrater reliability, Saito et al [15] recommend a higher number of ratings when the potential variance between ratings is high, 20 to 25 ratings were desired for each app. Therefore, with each judge providing up to 3 ratings, 17 apps were selected.

**Table 1:** List of apps and their modality or purpose.

App name	Modality or purpose
Headspace	Mindfulness and meditation
Lumosity	Cognitive training
iSleep Easy	Meditation and restful sleep
FitBrains	Cognitive training
Happify: For Stress & Worry	Well-being and happiness
Serenita	Stress and anxiety
SuperBetter	Goal setting, resilience, motivation
Flowy	Breathing and relaxation
The Mxindfulness App	Mindfulness and meditation
HAPPYneuron	Cognitive training
Smiling Mind	Mindfulness and meditation
Pacifica (now Sanvello)	Anxiety and depression
Virtual Hope Box	Anxiety and depression
Peak	Cognitive training
Personal Zen	Stress and anxiety
BrainHQ	Cognitive training
Wildflowers	Mindfulness and meditation

### Measures

#### Gamification Principles

A novel self-report measure was developed based on the gamification principles for internet interventions model. The measure is composed of five items, with each item assessing the principle of gamification. Items include a description of the gamification principle (eg, meaningful purpose) in question and instruct the rater to judge the presence of that principle within the intervention. Embedded within the descriptions are probing questions to help determine the extent the principle is present (eg, “Does the application allow the user to make decisions about how they reach their goal?”). One item was used to assess each gamification principle to increase the efficiency of raters and limit the response burden. Single-item scales have been used and validated to assess complex constructs such as self-esteem, job satisfaction, and personality traits [16-18]. The items were assessed on a 6-point Likert scale (0=complete absence of the gamification principle and 1-5=weak to strong presence of the principle in question; Multimedia Appendix 1).

#### App Quality (Mobile App Rating Scale)

The Mobile App Rating Scale (MARS; Stoyanov et al [19]) is a widely used measure of app quality that focuses on aspects of engagement, functionality, aesthetics, and information quality. MARS scores for each program included in this study were obtained from the PsyberGuide website. MARS scores are averaged from multiple independent raters who either have expertise in health interventions or psychology, technology development or design, or lived experience with intended clinical issues. Each MARS score was calculated using a combination of at least three raters. MARS scores are represented as *user experience* ratings (with a maximum score of 5.0) on the PsyberGuide website.

#### App Credibility (PsyberGuide Credibility)

PsyberGuide credibility ratings are meant to determine the likelihood that a given product will produce the proposed benefits. It is based on an assessment of the strength of research evidence, source of research evidence, specificity of the app, expertise of the development team, number of app store ratings, and recency of updates. PsyberGuide credibility rating scores are made by a team of trained reviewers consisting of undergraduate or masters-level students using an approval and consensus process (maximum score of 5.0). PsyberGuide credibility rating scores were obtained from the PsyberGuide website.

#### App Store Rating

The app store rating (Apple or Android) for each intervention was obtained. App store ratings are based on a system of stars (0-5), with a higher number of stars indicating a greater degree of liking the app.

### Procedure

Each of the 133 raters was randomly assigned to evaluate three apps. Raters were trained using a 2-part approach. The first was a 75-min training session that reviewed the theory of heuristic evaluations, a core concept in HCI. Heuristic evaluations occur when trained raters use a system and rate how well the design conforms to a set of described heuristics [20]. The heuristics used for this study were the five principles of gamification [9]. The training also involved teaching the raters about the principles of gamification, as these were the heuristics to be used for comparison when rating. Although this is not the prototypical use of a heuristic evaluation (a company, eg, would typically rate a user interface against a set of design principles), the training focused on how the process of rating gamification in apps was analogous to a classical heuristic evaluation. The apps were rated against a different set of heuristics (gamification principles), and these principles were enumerated and discussed in detail during the training. The training also provided a broad overview of digital health interventions (definition and brief examples) but did not include any detailed training in this area. To increase our confidence that the ratings were done thoughtfully and consistent with the training guidelines, raters were required to provide a written justification for their ratings.

Raters were given 2 weeks to evaluate their apps and were instructed to use each app for at least 15 min every day. Specifically, raters were asked to use the app as a normal user and to examine the presence of each of the gamification principles. Although rater usage was not tracked, the raters were encouraged to maintain lists of specific examples of each gamification principle they encountered and to list them in their written justifications. After 2 weeks, raters were given 1 week to complete a 10-question survey. For each gamification principle, raters were asked whether the principle was present in the app (binary), and, if so, to what degree that principle was present (1-5 scale). These pairs of questions were later combined to create single 6-point (0-5 scale) responses. For the presence questions (1-5 scale), a description was provided for scores 1, 3, and 5. This was done to allow raters some flexibility in interpreting the score between these endpoints and middle points. To ensure that raters had provided thoughtful responses, they were asked to provide a justification for each of their scores by writing a supporting paragraph. The raters were assigned a grade to complete this assignment and to provide reasonable and thoughtful justifications for the provided scores. Raters provided reasonable justifications, earning an average of 9.4 out of 10 on this assignment. The university institutional review board (IRB) was contacted with the details of this endeavor, and it was determined that no IRB protocol was necessary.

### Statistical Analysis Outliers

Statistical analysis outliers (ratings more than two SDs from the mean) were identified. However, on review of the justifications provided by these raters, no data were removed. For each program and survey question combination, the mean scores and SDs were calculated. Interrater reliability scores for each app were calculated to determine the degree of agreement among the independent raters. Interrater reliability was obtained by using the weighted Fleiss kappa [21,22] for each app, across all questions and raters. Fleiss kappa is recommended when there are more than two raters.

Correlations and *P* values were calculated between the average ratings of each gamification principle and each of the three dependent measures (ie, app quality, app credibility, app store rating). Correlations between the gamification principles were examined to detect the presence of collinearity among the ratings. A custom program, written in Python, was used for outlier identification, coalescing the raw data into mean (SD), and for computing the interrater reliability (ie, all results from Tables 2 and 3). The statistics program R was used to compute all correlation coefficients and related statistics (ie, all results from Multimedia Appendix 2 and Table 4).

**Table 2:** Mean and SD ratings for each gamification principle across raters.

App name	Meaningful purpose, mean (SD)	Meaningful choice, mean (SD)	Supporting player archetypes, mean (SD)	Feedback, mean (SD)	Visibility, mean (SD)
Headspace	4.29 (0.84)	3.92 (1.04)	3.50 (1.47)	3.33 (1.49)	4.08 (1.08)
Lumosity	4.36 (0.79)	3.08 (1.49)	3.40 (1.10)	4.32 (0.93)	3.76 (1.21)
iSleep Easy	3.92 (1.00)	2.71 (1.57)	0.17 (0.47)	1.17 (1.40)	0.25 (0.60)
FitBrains	4.10 (1.38)	2.76 (1.69)	3.33 (1.04)	4.38 (1.09)	4.29 (1.39)
Happify: For Stress & Worry	4.26 (1.07)	3.61 (1.31)	3.26 (1.72)	3.83 (1.27)	3.74 (1.36)
Serenita	3.91 (1.06)	2.57 (1.50)	1.43 (1.58)	3.43 (1.47)	3.70 (1.23)
SuperBetter	3.65 (1.05)	3.61 (1.34)	3.00 (1.41)	3.26 (0.94)	3.48 (1.25)
Flowy	1.95 (1.07)	1.64 (1.37)	1.91 (1.44)	2.32 (1.49)	2.91 (1.20)
The Mindfulness App	3.59 (1.34)	3.59 (1.30)	2.41 (1.44)	2.18 (1.61)	2.86 (1.42)
HAPPYneuron	4.15 (0.91)	3.25 (1.30)	2.90 (1.34)	3.35 (1.24)	3.55 (1.16)
Smiling Mind	4.09 (1.06)	3.78 (1.18)	2.57 (1.61)	3.26 (1.80)	3.96 (0.95)
Pacifica	4.04 (1.04)	4.40 (0.98)	4.56 (0.70)	3.64 (1.29)	4.16 (0.97)
Virtual Hope Box	3.30 (1.43)	3.43 (1.38)	2.43 (1.77)	0.70 (1.16)	0.17 (0.38)
Peak	4.35 (0.91)	3.13 (1.73)	2.96 (1.60)	4.26 (0.85)	4.65 (0.70)
Personal Zen	2.14 (1.12)	0.86 (1.17)	0.95 (1.13)	2.86 (1.36)	3.43 (1.37)
BrainHQ	4.08 (1.19)	2.54 (1.68)	2.58 (1.55)	4.00 (0.82)	3.75 (1.16)
Wildflowers	3.65 (1.34)	2.78 (1.44)	2.35 (1.68)	3.52 (1.35)	3.91 (1.10)

**Table 3:** Interrater reliability scores for each app.

App	Interrater reliability
Headspace	0.53
Lumosity	0.57
iSleep Easy	0.67
FitBrains	0.55
Happify: For Stress & Worry	0.51
Serenita	0.53
SuperBetter	0.54
Flowy	0.52
The Mindfulness App	0.51
HAPPYneuron	0.54
Smiling Mind	0.52
Pacifica	0.58
Virtual Hope Box	0.61
Peak	0.57
Personal Zen	0.58
BrainHQ	0.54
Wildflowers	0.51

**Table 4:** Correlation matrix between gamification principles across all rated apps.

Gamification principle	Meaningful purpose	Meaningful choice	Supporting player archetypes	Feedback	Visibility
Meaningful purpose	N/A^a^	0.71	0.50	0.48	0.30
Meaningful choice	N/A	N/A	0.69	0.11	0.11
Supporting player archetypes	N/A	N/A	N/A	0.57	0.57
Feedback	N/A	N/A	N/A	N/A	0.92
Visibility	N/A	N/A	N/A	N/A	N/A

^a^N/A: not applicable.

## Results

### Overview

The means and SDs for each gamification principle across the 17 apps are shown in Table 2. There was a wide degree of variation in gamification present in the apps. The average gamification score (ie, the mean of all five gamification principle ratings) ranged from 1.64 to 4.16 out of 5. Among the principles, supporting player archetypes was judged as being the least present (average 2.57 out of 5), whereas meaningful purpose was judged as being most present (average 3.75 out of 5).

Table 3 lists the interrater reliability scores for each app studied. Interrater reliability scores range from 0.51 to 0.67, indicating acceptable levels of agreement across raters for each app [23].

### Criterion Validity and Associations Between Gamification Principles and Other Measures

Table 5 lists the app credibility score (ie, PsyberGuide Credibility Score), the MARS score, and the app store ratings for each app. One app (HAPPYNueron) did not have an app store rating. In general, the three scores were not strongly associated with one another (*r*=−0.09 for PsyberGuide Credibility vs MARS; *r*=0.32 for MARS vs app store rating; *r*=0.04 for PsyberGuide Credibility vs app store rating), suggesting that each of these three measures likely represent different aspects of app quality.

**Table 5:** Various metrics scoring each studied app (1-5 scale).

App	PsyberGuide Credibility Score	Mobile App Rating Scale	App store rating
Headspace	4.64	4.74	4.9
Lumosity	3.21	4.34	4.7
iSleep Easy	3.55	3.01	4.6
FitBrains	2.85	4.67	3.7
Happify: For Stress & Worry	3.92	3.34	4.5
Serenita	3.2	3.2	3
SuperBetter	3.55	4.39	4.7
Flowy	2.5	4.1	4.3
The Mindfulness App	2.85	3.3	4.4
HAPPYneuron	2.5	4.15	N/A^a^
Smiling Mind	2.85	4	4.6
Pacifica	2.85	4.7	4.7
Virtual Hope Box	3.92	3.59	4.4
Peak	2.85	4.52	4.7
Personal Zen	3.95	3.77	2.6
BrainHQ	4.6	4.11	4.6
Wildflowers	2.85	4.08	4.33

^a^N/A: not applicable.

Multimedia Appendix 1 lists the correlation coefficients as well as *t* test statistics (2-tailed) and *P* values among gamification principles ratings and the PsyberGuide Credibility Score, the MARS rating, and the app store rating. There were generally weak correlations between the gamification principle ratings and the PsyberGuide Credibility Score, indicating a low degree of overlap. Supporting player archetypes (*P*=.001), feedback (*P*=.01), and visibility (*P*=.008) correlated strongly with the MARS rating, whereas meaningful purpose (*P*=.04), meaningful choice (*P*=.002), and supporting layer archetypes (*P*=.04) correlated strongly with the app store ratings. A closer examination of the significant associations between the gamification principles and the MARS and app store ratings revealed between 25% and 52% shared variance (r-squared) among these variables, indicating that they are related yet measure different constructs.

### Inter-Relationships Between Gamification Principles

Table 4 contains a correlation matrix of the relationships among gamification principles. The strength of associations varied widely (*r*=0.11 to 0.92), with visibility and feedback as the most strongly associated and overlapping principles. On average, the correlations were *r*=0.50, indicating that principles were related yet separate from one another.

## Discussion

### Principal Findings

This study provides empirical support for the model of the five gamification principles for internet interventions. We believe this model will help researchers develop new interventions and evaluate existing interventions that better engage users through the proper implementation and integration of gamification techniques. By evaluating the gamification principles in 17 health apps, the findings from this study indicate that the gamification principles are not redundant with existing app measures.

A weak relationship was found between the gamification principle ratings and the PsyberGuide credibility score. The PsyberGuide credibility score is based on several factors, some of which have no intuitive relationship to the gamification principles evaluated in this study. For example, one aspect of the PsyberGuide credibility score involves the amount of research funding the app had garnered, which has no direct connection with gamification. Other aspects of the PsyberGuide credibility score focus on the degree to which research is available on the efficacy of that app or the frequency of the updates to the app. Although some of these aspects, such as the frequency of updates, have been found to be useful predictors of some evaluations of apps such as expert-rated quality or user ratings [24], they would not be expected to categorize the features into the app. In sum, the lack of relationship between the credibility score and the gamification principle ratings suggests that the credibility of an app (which includes efficacy as well as other issues such as software support, input from experts, etc) is largely independent of its level of gamification.

There were significant relationships between 3 of the 5 gamification principles (supporting player archetypes, feedback, and visibility) and the MARS score. Theoretically, one would imagine some overlap between our gamification model and user experience aspects such as engagement. The MARS (collected from PsyberGuide) [19] explicitly mentions qualities that overlap with feedback and visibility, namely, items such as *quality or quantity of information* or *visual information*. However, the gamification principles go beyond the MARS by providing specific guidelines for presenting this information and contextualizing it within a user’s broader goals and understanding. Thus, even with some conceptual overlap, the gamification principles still have added value. Engagement and attrition have long been identified as an issue within eHealth [25-27], and this can be helped by having patients play a more active role in their own care [28]. Gamification principles may therefore facilitate the execution of game mechanics by providing researchers another avenue to explore and measure engaging features that involve the patient. Similarly, the gamification principle of *supporting player archetypes* has an intuitive overlap with MARS items involving engagement and subjective quality. However, the gamification principle of supporting player archetypes presents specific mechanisms through which these qualities can be achieved and are commonly done in games and game-like systems. Thus, we believe that our gamification principles are not in direct conflict with the MARS; rather, they provide a roadmap for ways to increase app quality.

Three different gamification principles (meaningful purpose, meaningful choice, and supporting layer archetypes) were significantly associated with the app store ratings. In contrast to the MARS, the app store ratings are single overall ratings provided by end users. By directly sampling from end users, the app store rating may be viewed as largely a reflection of user choice. There was a strong association between app store ratings and the gamification principle of meaningful choice (*r*=0.71), suggesting that users may value having agency in how they use and navigate through an app. App store ratings are subjective, personal, and nonstandardized and have been shown to be an indication of app popularity, but not clinical outcomes [29]. Thus, it is notable that the strongest related principles of meaningful purpose (the user has a goal in using the app), meaningful choice (the user has agency over their progress), and supporting player archetypes (the app leverages individual user characteristics) all directly involve the user, whereas the other two gamification principles, feedback and visibility, relate more specifically to the app design, its presentation, and organization of information.

This study extends the existing literature that aims to understand technology-based behavioral interventions by identifying and coding their features [24,30,31]. Although past work has used different conceptual models, this study evaluates features based on gamification principles. Our findings that gamification principles overlap with some, but not all, other assessments of app quality and popularity indicate both convergent and discriminant validity of the gamification principles assessment. Future work may wish to apply this assessment to other behavioral change interventions and examine if gamification principles in apps correlate with real-world engagement or effectiveness. In addition, researchers and developers would be well served with a streamlined evaluation method for incorporating gamification (or measuring the presence of gamification) in their apps. Future work should focus on providing such artifacts and continuing to study their utility.

### Strengths and Limitations

Several potential limitations exist for this study. Most notably, although the raters were trained with several modes of material, they were undergraduate students and not experts; however, several results from the study help limit this concern. The interrater reliability scores (weighted kappa) were all within the acceptable range (κ>0.50), which suggests that although there was variance in the scores, the raters generally gave similar ratings to apps. In addition, raters provided written justifications for each of their scores. An expert read through these justifications with the intention of removing ratings that included clear evidence of a poor rating. In the end, no ratings were deemed to lack sufficient justification, and all ratings were included in the analysis presented here.

Owing to the difficulty in calculating internal consistencies and establishing construct validity for single-item measures (to assess each gamification principle), future work should examine ways to assess gamification using more items. Although our decision was influenced by a desire to reduce the burden on raters and increase the efficiency with which ratings could be completed, there are many ways to assess gamification that should be explored in future work. However, because the results show evidence of both convergent and discriminant validity, there is some evidence that construct validity does exist.

Potential bias also exists with the app selection methodology. Although the PsyberGuide listing likely contained more than 17 apps that fit the inclusion criteria, this was deemed a sufficient number, given the quantity of raters and associated ratings to be obtained. An attempt was made to include a heterogeneous sample of apps that covered a variety of areas of focus and population types. However, a more systematic approach could have been used to select the apps, or, with sufficient resources, to include all apps.

There were some strong associations among gamification principles. Most notably, the principles of feedback and visibility had a strong relationship (*r*=0.92), potentially suggesting that these principles may measure the same underlying construct. It is possible that although these principle definitions are indeed mutually exclusive (ie, feedback involves the effects of user actions on the future, whereas visibility shows the results of accomplishments from the past), perhaps apps tend to use them in unison as they complement one another. It is also possible that the raters did not fully understand the distinction between these principles and may have conflated them.

Strong correlations occurred among other gamification principle ratings as well, most of which are less easily explainable. For example, the principles of meaningful choice and meaningful purpose correlate strongly (*r*=0.71), suggesting raters may have interpreted the meaningful qualifier as being shared across the ratings (ie, to make a meaningful choice, there must be a meaningful purpose toward which the user is progressing). More research is necessary to determine the nature of these correlations. In addition, no analysis was done to compare apps of similar purpose (eg, comparisons within mindfulness apps). Other studies have focused on comparing MARS scores or other measures among similar apps [32-35]. There might exist patterns that are stronger or weaker within apps of a specific purpose that could provide additional insight into the role of gamification and other measures (credibility rating, MARS, and app store rating), and this might be an avenue for future research.

### Conclusions

In short, this paper is the first evaluation of a method determined to assess previously proposed gamification principles [9]. Our findings suggest that gamification principles relate to some, but not all, previously proposed methods of assessing app quality and popularity, which blend ratings made by experts (such as the PsyberGuide credibility scores), ratings made by consumers (app store ratings), and ratings made by both (in this case, the MARS scores). The pattern of relationships has considerable face validity, including those with the MARS scores and user ratings, and the lack of relationship with PsyberGuide credibility scores. This lack of rating does not indicate that either scale is invalid, but instead that the rating of gamification principles and credibility might offer unique perspectives in terms of understanding apps. The demonstration of a process of rating these products through collaborative assessments of a team of lightly trained raters also demonstrates a potential way to easily and scalably understand the growing number of technology-based behavioral interventions that are being developed. We also believe the demonstration that gamification principles have value helps support the application of these principles to the design of novel technology-based behavioral interventions and might help developers incorporate evidence-based behavior change strategies into their products. As such, both the methodological and conceptual contributions of this work can move forward the research and practice of gamification principles being thoughtfully applied to behavior change digital interventions.

## Appendix

Multimedia Appendix 1 Gamification Principles and associated questionnaire used by the raters in this study.

Multimedia Appendix 2 Correlation coefficients, t test statistics, and P values between each gamification principle’s average ratings and each of the PsyberGuide credibility rating, Mobile App Rating Scale, and app store rating.

## Abbreviations

eHealth: electronic health
HCI: human-computer interaction
IRB: institutional review board
MARS: Mobile App Rating Scale
mHealth: mobile health
