# Supplementary material for: The Model of Gamification Principles for Digital Health Interventions: Evaluation of Validity and Potential Utility
Source: J Med Internet Res. 2020 Jun 10;22(6):e16506. doi: 10.2196/16506 (PMC7315368; doi:10.2196/16506)
Supplement: Multimedia Appendix 1 [file jmir_v22i6e16506_app1.pdf]

GAMIFICATION PRINCIPLES AND SCALE

Meaningful Purpose

Short Description

The application presents a purpose that is meaningful to its users and the mechanics of the application amplify and enforce this meaning.

Related Questions

Does the app present a purpose that is meaningful to the user?

Do the mechanics / features of the application enhance this purpose?

Do the mechanics / features of the application directly relate to the user's primary purpose?

Questionnaire Item

To what degree is meaningful purpose present in this application?

0	Meaningful Purpose is not present in this application
1	The application does not have a clear meaningful purpose for users and the features of the app reinforce this
3	The application has a clear meaningful purpose for users but several features distract from or don't explicitly enhance that purpose
5	The purpose for which users use this app is meaningful to them, and the features of the app consistently reinforce and focus on this purpose

Meaningful Choice

Short Description

The application gives users agency over how they achieve their goals

Related Questions

Does the application allow the user to make decisions about how they reach the goal?

If so, how meaningful are these decisions? Is the correct choice obvious, or are there legitimate distinct paths to success?

Questionnaire Item

To what degree is meaningful choice present in this application?

0	Meaningful Choice is not present in this application
1	The application does not provide the users with any meaningful choices that affect their path towards the goal
3	The users make choices that affect their progress towards goals, but the choices are either not substantial enough to be interesting or are trivial choices to make
5	The application provides meaningful choices that affect progress towards the goal and these choices lead to interesting and unique successful paths through the application

Supporting Player Archetypes

Short Description

The application implements mechanics that leverage individual player characteristics

Related Questions

Can players use the app “their way”?

Are their achievement based features (badges, achievements, etc.)

Are their social features?

Are their exploration based features?

Questionnaire Item

To what degree does the application Support Player Archetypes?

0	The application does not support player archetypes
1	The app does not support even one player archetype. There are no achievement systems, no social mechanics, and no room for exploration.
3	The app presents features that support at least one archetype (perhaps achievers through badges or similar) but there is not much variation in how the system can be used.
5	The app presents features that support different player types and different styles of play. Users are able to use the app in myriad of ways depending on their personal preferences and style.

Feedback

Short Description

The application communicates precisely how player actions affect progress towards the goal

Related Questions

Does the app make it very clear how your actions are affecting your path towards the goal.

When you do things, is it obvious how much that helped you reach your goal? Do you know why?

Are you given enough feedback that you can plan your path to your goal somewhat effectively?

Questionnaire Item

To what degree does the user receive Feedback when using the application

0	The user does not receive Feedback when using this application
1	The user receives little to no feedback, rendering the user clueless as to how goals are achieved and how their actions might lead to success.
3	The user receives regarding how their actions affect their goals. However, there is noticeable amounts of confusion regarding how goals can be directly attained.
5	The user consistently receives feedback that directly communicates how the user's actions have led to progress towards their goals. There is no noticeable lack of feedback.

Visibility of Progress

Short Description

The application reminds players how much progress has been made and how much more is needed

Related Questions

Does the app clearly provide access to a history of the user’s progress?

Is this progress clear and understandable?

Is this progress motivating?

Questionnaire Item

To what degree does the application present Visibility of the user's progress

0	The application does not present the user with Visibility of their progress
1	The app contains no (or almost no) visibility of progress. Users have little to no means of recalling the progress they have made over their time using the application.
3	Users have the ability to view their progress but this could be noticeably Improved. Perhaps the feature(s) are difficult to find/use or do not present as much information as they could.
5	Users have clear and ready access to an understandable and visible view of the progress they have made. This include immediate progress (e.g., one day) and global progress (e.g., since installing the app)
